# Supplementary material for: Serological analysis in humans in Malaysian Borneo suggests prior exposure to H5 avian influenza near migratory shorebird habitats
Source: Nat Commun. 2024 Oct 17;15:8863. doi: 10.1038/s41467-024-53058-y (PMC11487116; doi:10.1038/s41467-024-53058-y)
Supplement: Supplementary file 1 — Supplementary Information [file 41467_2024_53058_MOESM1_ESM.pdf]

Supplemental Information

1. Wet laboratory study procedures and overview

Supplemental Figure 1. Overview of experimental procedures and study design. **a**. We began by randomly selecting 500 individuals from each of the four districts sampled in the Sabah state from samples collected between September 17, 2015 and December 12, 2015 for Fornace et al[1]. Antibody binding of haemagglutinin in these individuals was tested by ELISA. A cohort of Scottish blood donors from 2020 was used as a control cohort which was thought to have seasonal HA exposure but were presumed to be H5 negative. The urban Malaysian cohort from Kota Kinabalu, Malaysia was used to understand local baseline binding to H5N1 HA. These individuals were presumed to have less contact with livestock and wild migratory shorebirds than the rural cohort of 2,000 samples. The top 1% of rural Malaysian responders to H5N1 HA by ELISA were tested for neutralisation of three H5N1 HA pseudoviruses (along with a random selection of rural Malaysian samples and the Scottish blood donor cohort). Eight of the rural Malaysian neutralising samples were tested via a cross-reactivity depletion assay. **b.** Sampled villages and district locations in Sabah. Map generated in QGIS 3.30.2[2] using data from the Humanitarian Data Exchange (United Nations Office for the Coordination of Humanitarian Affairs)[3]. Natural Earth raster map data was used to generate the canvas map. **c.** Demographic data on sample cohort and samples selected for this study.

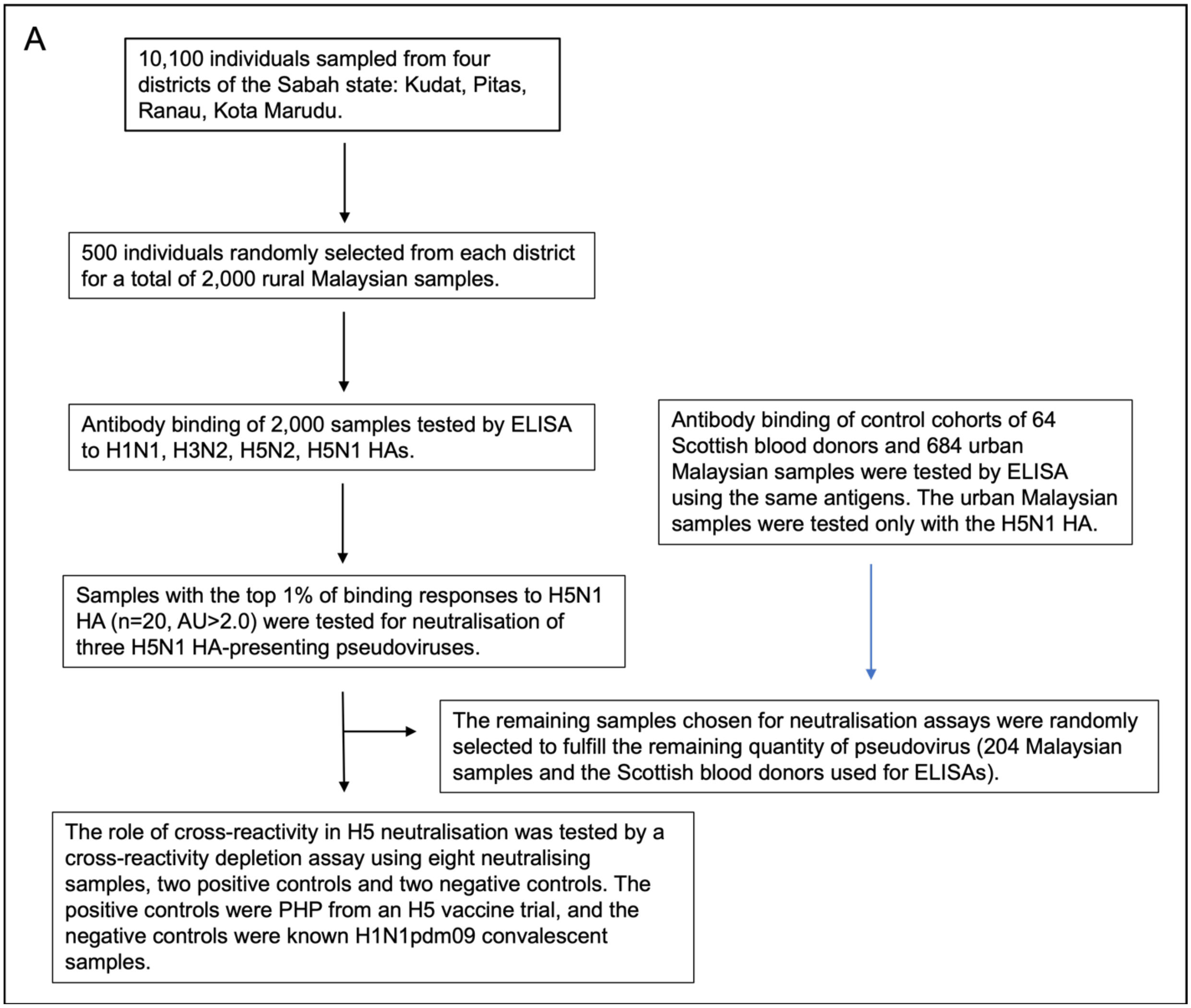
A
10,100 individuals sampled from four districts of the Sabah state: Kudat, Pitas, Ranau, Kota Marudu.
500 individuals randomly selected from each district for a total of 2,000 rural Malaysian samples.
Antibody binding of 2,000 samples tested by ELISA to H1N1, H3N2, H5N2, H5N1 HAs.
Antibody binding of control cohorts of 64 Scottish blood donors and 684 urban Malaysian samples were tested by ELISA using the same antigens. The urban Malaysian samples were tested only with the H5N1 HA.
Samples with the top 1% of binding responses to H5N1 HA (n=20, AU>2.0) were tested for neutralisation of three H5N1 HA-presenting pseudoviruses.
The remaining samples chosen for neutralisation assays were randomly selected to fulfill the remaining quantity of pseudovirus (204 Malaysian samples and the Scottish blood donors used for ELISAs).
The role of cross-reactivity in H5 neutralisation was tested by a cross-reactivity depletion assay using eight neutralising samples, two positive controls and two negative controls. The positive controls were PHP from an H5 vaccine trial, and the negative controls were known H1N1pdm09 convalescent samples.

B

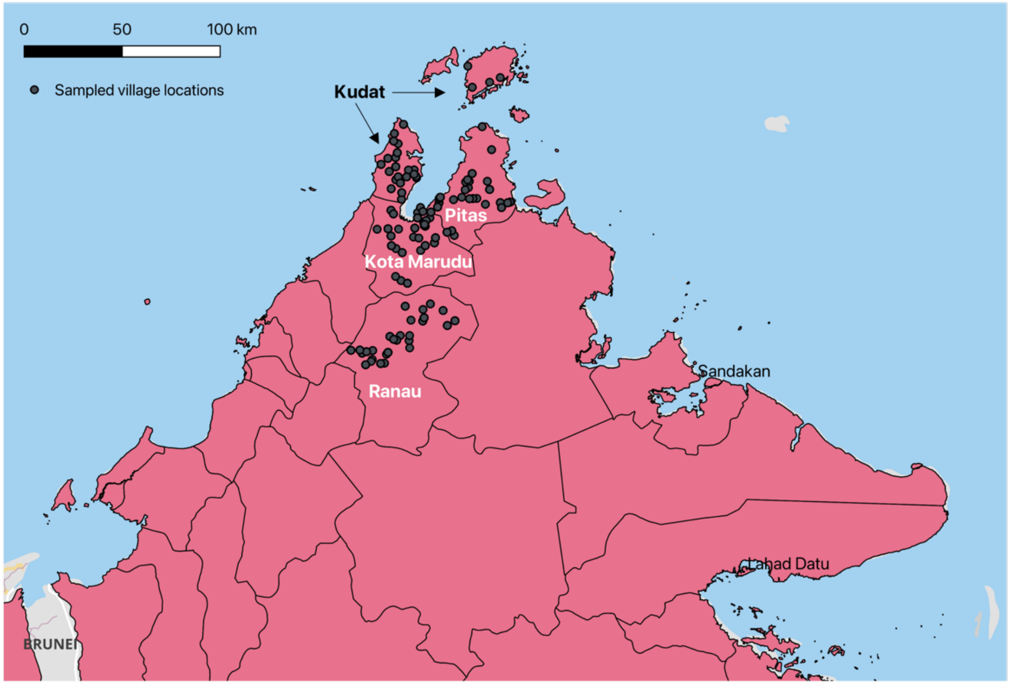

C

	Number of samples	Mean age and range	Gender
Total sample cohort (rural Malaysian)	10,100	29 (3 months-105 years)	47% male, 53% female
Total sample cohort used in this study (rural Malaysian)	2,000	29 (5 months- 97 years)	45% male, 55% female
Kudat: samples used in this study	500	29 (11 months- 87 years)	52% male, 48% female
Pitas: samples used in this study	500	27 (3 months- 92 years)	44% male, 56% female
Ranau: samples used in this study	500	27 (1-97 years)	45% male, 55% female
Kota Marudu: samples used in this study	500	31 (3 months- 94 years)	51% male, 49% female

2. Phylogenetic analysis of hemagglutinins

Supplemental Table 1. Phylogenetic analysis of hemagglutinins used in this study. Sequences were obtained from GISAID and aligned with the MUSCLE algorithm in MEGA. Pairwise distances were computed as the number of amino acid differences. The clade of each HA is included in parenthesis along with the use of each HA (either recombinant proteins used in ELISAs or pseudovirus used in microneutralisation assays).

	1	2	3	4	5
1. A/Indonesia/05/2005 HA (Clade 2.1.3.2) (Pseudovirus)					
2. A/Bar-headed goose/Qinghai/1A/2005 HA (Clade 2.2) (Pseudovirus)	22.00				
3. A/chicken/Malaysia(Sabah)/6123/2018 (Clade 2.3.2.1c) (Pseudovirus)	43.00	39.00			
4. A/Duck/Laos/3295/2006 HA (Clade 2.3.4) (ELISA)	20.00	18.00	36.00		
5. A/snow goose/Missouri/CC15-84A/2015 HA (2.3.4.4) (ELISA)	50.00	44.00	48.00	38.00	

3. Comparison of ELISA results

Supplemental Figure 2. Correlation matrix of ELISA binding results. Spearman's rank correlation coefficients (r) were calculated in GraphPad Prism 10.0.3. Correlation coefficients range from -1 to 1, where 1 indicates positively correlated data and -1 indicates negatively correlated data. An r of 0 indicates the ELISA values do not vary together. **a** displays the correlations of ELISA binding in the rural Malaysian cohort (n=2000). **b** shows the correlation matrix of ELISA binding results for the Scottish blood donor cohort (n=63).

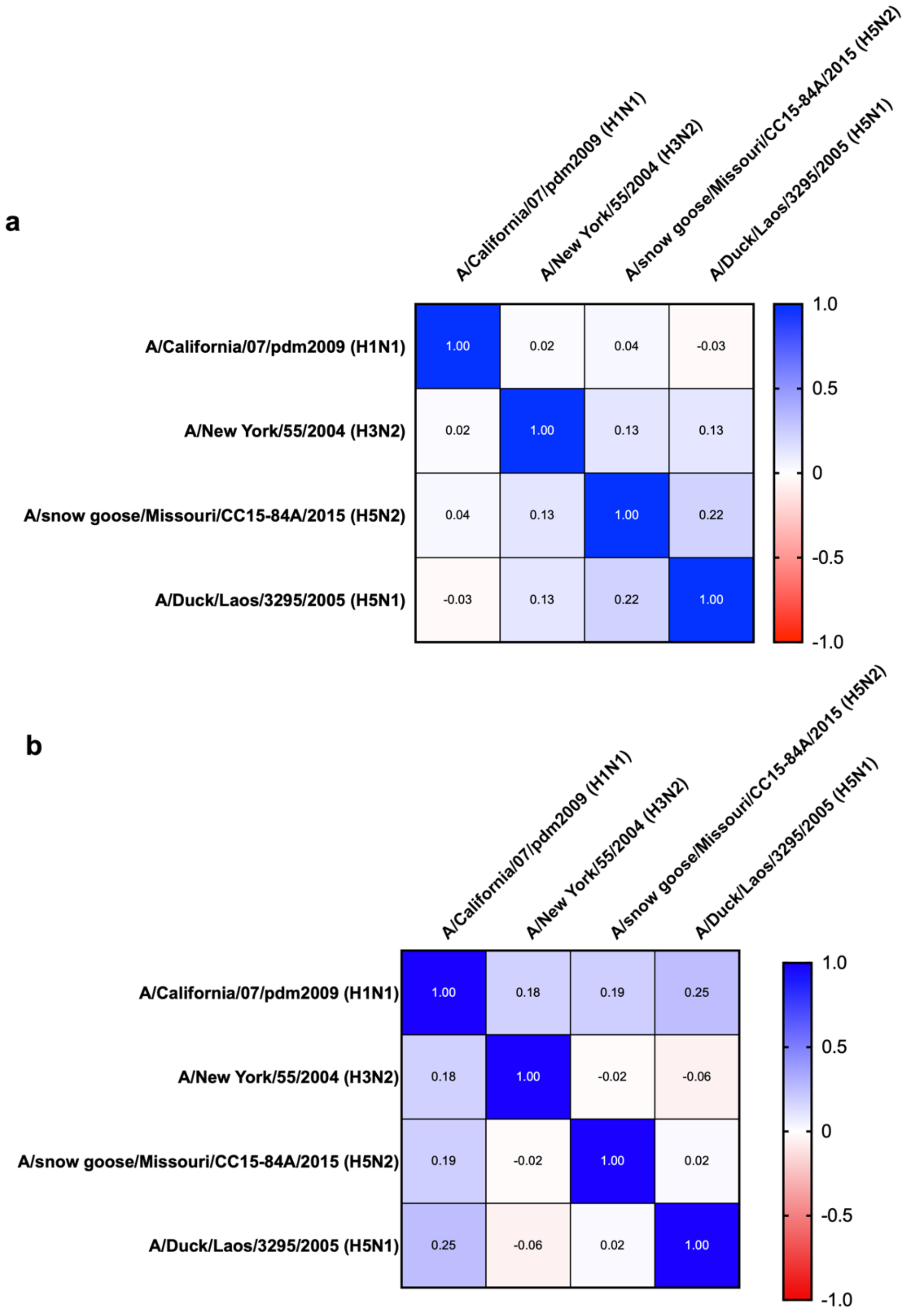

4. Neutralisation assay optimisation

Supplemental Figure 3. Creation of seropositive cut-offs for pseudotyped neutralisation assays. NT50 is the 50% neutralising titre, which is represented here as the dilution factor of the serum necessary to reach 50% inhibition of pseudovirus growth. The highest Scottish blood donor pseudoneutralisation NT50 value was used to create a cut-off, as these individuals were considered unlikely to have been exposed to a real H5N1 virus as in Thompson et al[4]. **a** 14 samples were considered seropositive to A/Bar-headed goose/Qinghai/1A/2005(H5N1). **b** 3 samples were considered seropositive to A/Indonesia/05/2005(H5N1). **c** 12 samples were considered seropositive to A/Chicken/Malaysia(Sabah)/6123/2018(H5N1).

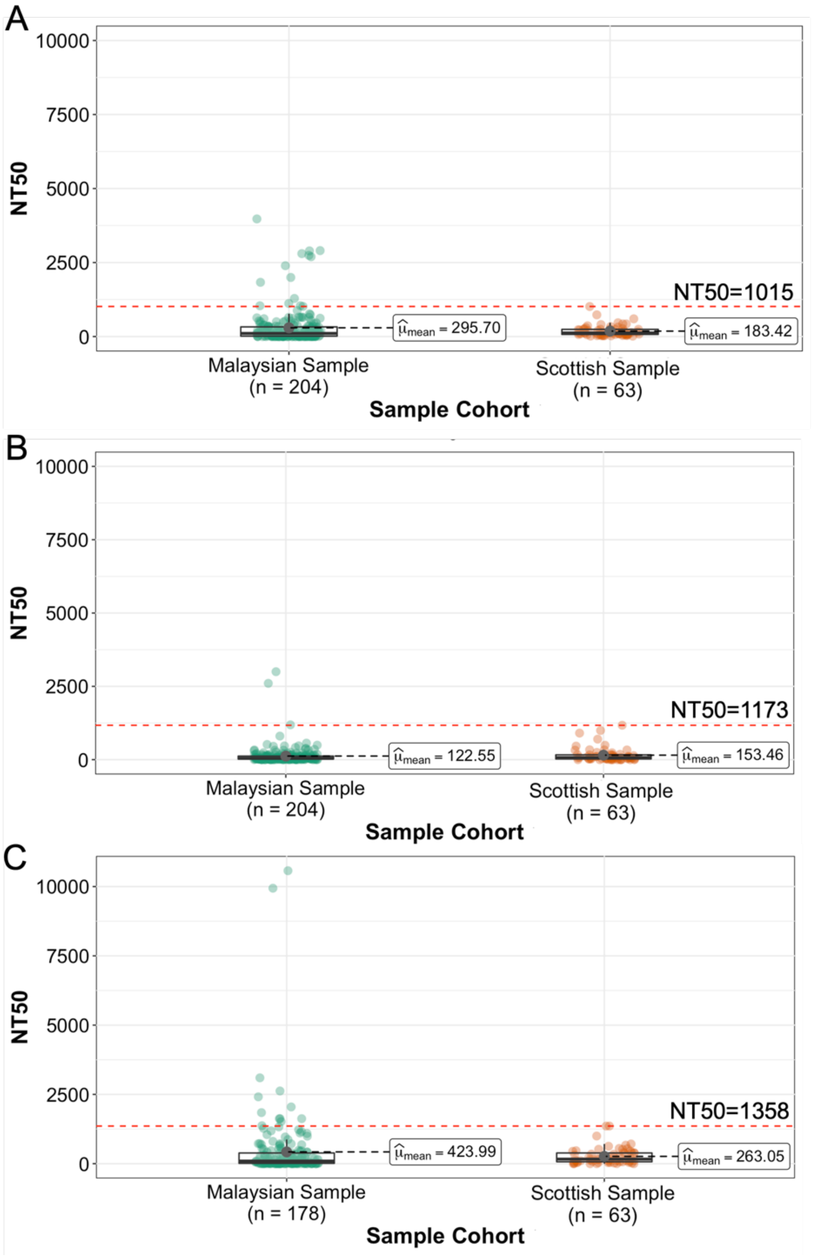

5. Confirmation of bead-antigen conjugation

Supplemental Figure 4. Confirmation of bead-antigen conjugation by flow cytometry. **a** Side scatter versus forward scatter plots were used to draw gate 1 to exclude debris and doublets. **b** The count versus PE histogram was used to observe PE fluorescence in the different conditions and shows the contents of gate 1. Data was analysed with FlowJo™ 10.8.2. H1 CA coated beads indicates A/California/07/2009(H1N1) HA, H1 NY denotes A/New York/18/2009(H1N1) HA, and H3 represents A/Wisconsin/67/2005(H3N2) HA.

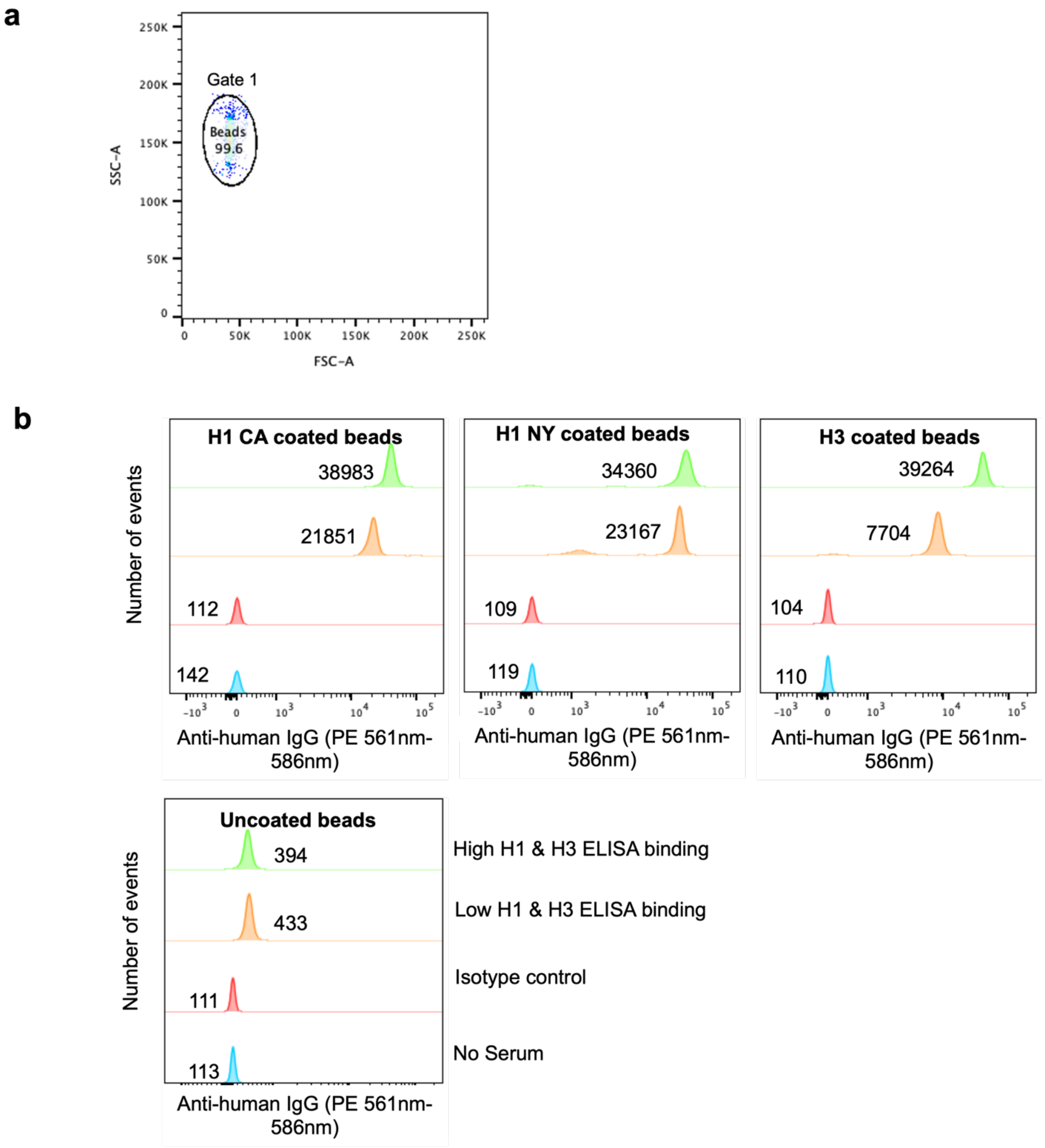

6. ELISA control cohorts and plate controls

Supplemental Figure 5. Binding to A/Duck/Laos/3295/2005(H5N1) HA by ELISA including control cohorts and plate controls. Plates were coated with 0.125μg/mL of A/Duck/Laos/3295/2006(H5N1) HA in 50μL 1X PBS. All samples shown were tested in duplicate at a 1:50 dilution. Rural Malaysia indicates our cohort of interest for this study. The Urban Malaysia cohort indicates samples collected from blood donors in Kota Kinabalu, Malaysian Borneo. Scottish indicates samples from the Scottish blood donor cohort. PHP post H5 vaccination refers to pooled human plasma from an H5 vaccination trial, obtained from BEI Resources. One H5 PHP pool was considered medium titre, and the other pool was low titre according to the suppliers, hence the two distinct points. These individuals were immunized intramuscularly with two doses of an inactivated monovalent subvirion investigational influenza using A/Indonesia/05/2005(H5N1). The Oxford and UMS plate controls are polyclonal goat antiserum raised against our ELISA antigen. **a** Shows age-matched cohorts, where the ages range from 18-65. **b** Shows the full rural and urban Malaysia cohorts, ages ranging from 0-104 with all controls.

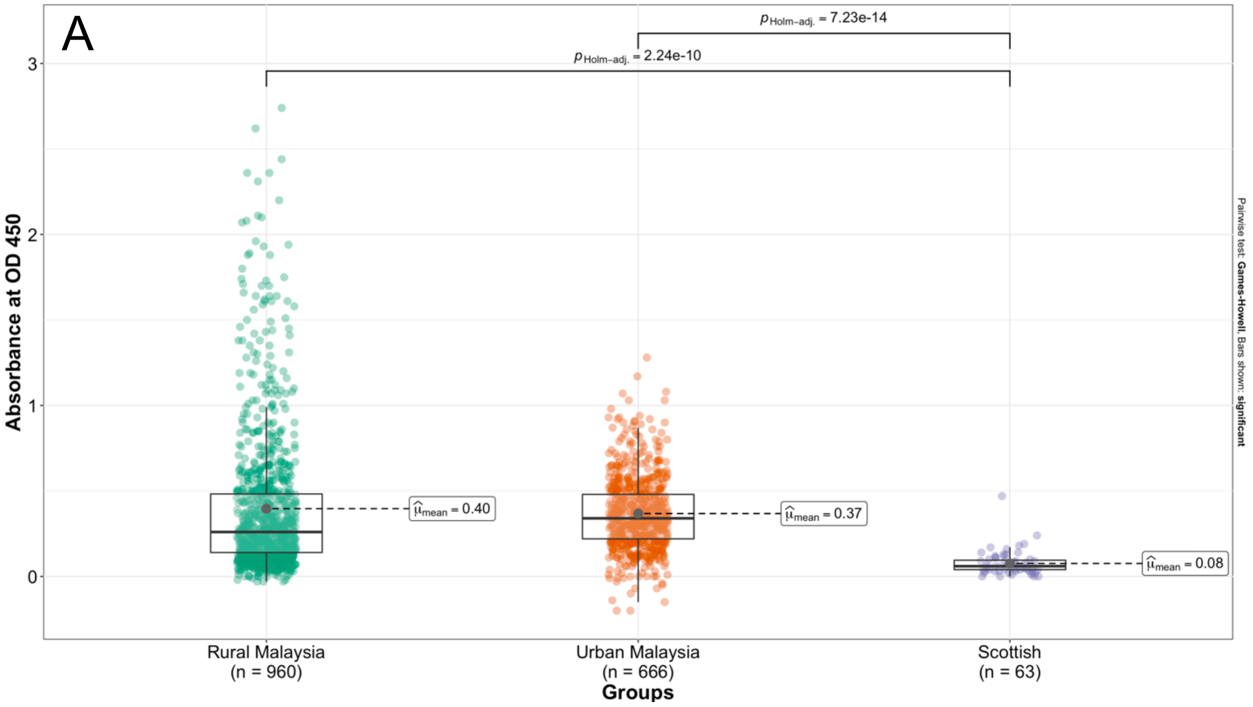

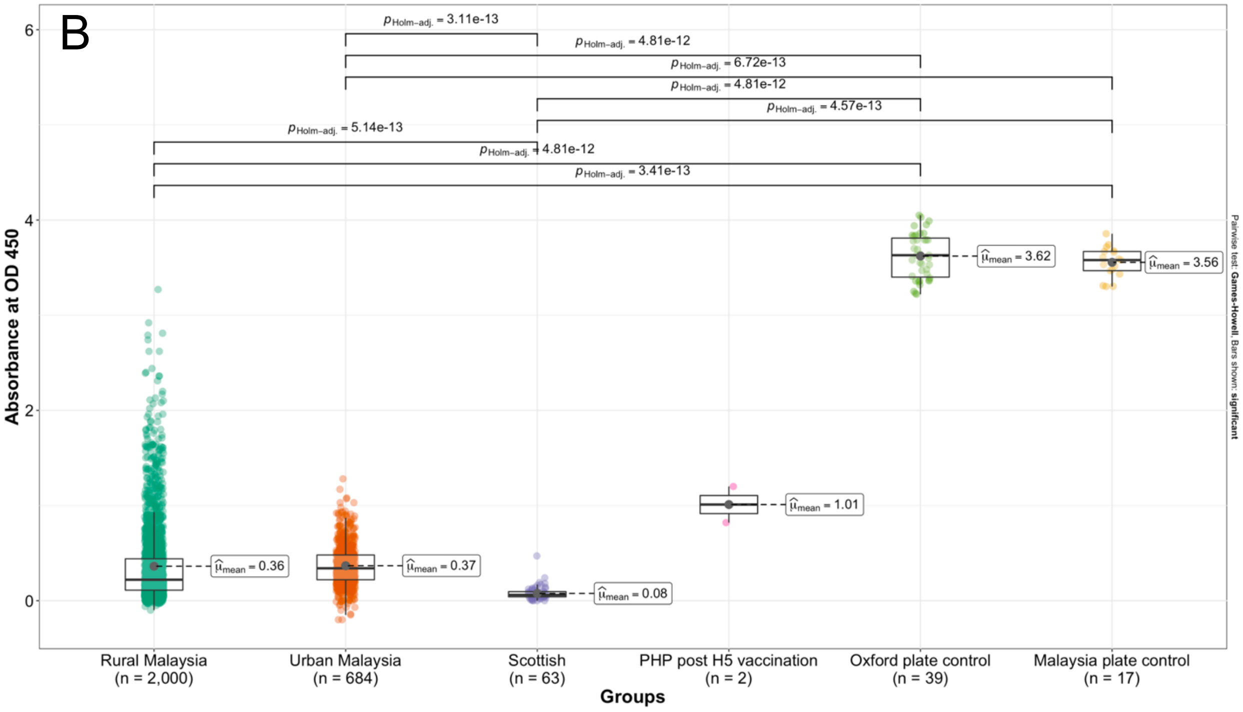

B
$p_{Holm-adj.}$ = 3.11e-13
$p_{Holm-adj.}$ = 4.81e-12
$p_{Holm-adj.}$ = 6.72e-13
$p_{Holm-adj.}$ = 4.81e-12
$p_{Holm-adj.}$ = 4.57e-13
$p_{Holm-adj.}$ = 5.14e-13
$p_{Holm-adj.}$ = 4.81e-12
$p_{Holm-adj.}$ = 3.41e-13
Absorbance at OD 450
$\hat{\mu}_{mean}$ = 0.36
$\hat{\mu}_{mean}$ = 0.37
$\hat{\mu}_{mean}$ = 0.08
$\hat{\mu}_{mean}$ = 1.01
$\hat{\mu}_{mean}$ = 3.62
$\hat{\mu}_{mean}$ = 3.56
Rural Malaysia (n = 2,000)
Urban Malaysia (n = 684)
Scottish (n = 63)
PHP post H5 vaccination (n = 2)
Oxford plate control (n = 39)
Malaysia plate control (n = 17)
Groups
Pairwise test: Games-Howell. Bars shown: significant

7. Geostatistical analysis of H5 binding and wild distributions

Hierarchical models of environmental risk factors were integrated into a Bayesian framework with the Matérn covariance function used as a spatial effect as in Fornace et al[1]. and Klim et al[5]. The deviance information criterion (DIC) was used as a method to compare our models. A smaller DIC is indicative of improved model fit. The DIC for the model of wild bird distribution with a spatial effect and fixed effects (the environmental risk factors) was 1098.52, versus 5104.99 for the model with only a spatial effect, and 1526.31 for the null model. In the models predicting the probability of high H5 binding, the model with a spatial and fixed effect was also the best fit as the DIC was 378.30 (388.69 = spatial effect only, 8762.31= null model).

Supplemental Table 2. Multivariate environmental risk factor analysis of H5N1 binding distribution. Adjusted odds ratios for fixed effects which impact the odds of H5 binding being greater than 2 AU. The model with lowest AIC is shown (336.12). The log likelihood was -159.0579. MASS::stepAIC was used to determine the model with the lowest AIC using a stepwise parsimonious approach (both forward and backward selection)[6]. Data was mean centred and scaled prior to analysis. Odds ratios were calculated and plotted using the sjPlot[7] package in RStudio[8,9]. 95% confidence intervals (CI) indicate uncertainty around the adjusted odds ratios, while p-values indicate the statistical significance of the odds ratio (i.e. whether inclusion of the predictor variable significantly impacts the odds of H5N1 HA binding being greater than 2 AU). Bold p-values indicates that inclusion of a variable is statistically significant with p <0.05. P-values for the risk factors for the odds of H5N1 binding are as follows 3.33×10^{-7} (model intercept), 0.018 (normalized differential vegetation index), 3.93×10^{-6} (elevation in meters above sea level), 1.67×10^{-6} (distance from the sea in meters), 4.79×10^{-5} (mean diurnal range in °C), 4.85×10^{-7} (minimum temperature of coldest month in °C), 0.04 (precipitation of the wettest month in mm), 0.084 (precipitation seasonality), and 0.027 (distance from irrigated farmland in metres).

Environmental risk factors	Odds of H5N1 binding > 2 AU		
Predictors	*Adjusted odds ratios*	*95% CI*	*p-value*
(Intercept)	0.00	0.00 – 0.00	**<0.001**
Normalized differential vegetation index	1.66	1.10 – 2.57	**0.018**
Elevation (meters above sea level)	0.00	0.00 – 0.00	**<0.001**
Distance from the sea (m)	894.78	56.09 – 16572.80	**<0.001**
Mean diurnal range, 1970 - 2000 (°C)	0.00	0.00 – 0.00	**<0.001**
Minimum temperature of coldest month, 1970 - 2000 (°C)	0.00	0.00 – 0.00	**<0.001**
Precipitation of the wettest month. 1970 - 2000 (mm)	0.40	0.15 – 0.88	**0.040**
Precipitation seasonality, 1970 - 2000 (coefficient of variation)	5.38	0.83 – 38.44	0.084
Distance from irrigated farmland (m)	0.61	0.38 – 0.92	**0.027**

Supplemental Table 3. Multivariate environmental risk factor analysis of wild bird distribution. Adjusted odds ratios for fixed effects which impact the odds of a wild bird sighting. The model with lowest AIC is shown (316.2). The log likelihood was -150.1009. MASS::stepAIC was used to determine the model with the lowest AIC using a stepwise parsimonious approach (both forward and backward selection)[6]. Data was mean centred and scaled prior to analysis. Odds ratios were calculated and plotted using the sjPlot[7] package in RStudio[8,9]. 95% confidence intervals (CI) indicate uncertainty around the adjusted odds ratios, while p-values indicate the statistical significance of the odds ratio (i.e. whether inclusion of the predictor variable significantly impacts the odds of wild bird sightings). Bold p-values indicates that inclusion of a variable is statistically significant with $p < 0.05$. P-values for the risk factors for the odds of wild bird sightings are as follows 3.07×10^{-6} (model intercept), 6.88×10^{-10} (Euclidean distance from roads), 0.001 (elevation in meters above sea level), 0.001 (distance from the sea in meters), 6.90×10^{-5} (maximum temperature of warmest month in °C), 4.68×10^{-9} (precipitation of the wettest month in mm), 0.094 (distance from bush forest in meters) and 0.01 (distance from old forest in meters).

Environmental risk factors	Odds of wild bird sightings		
Predictors	*Adjusted odds ratios*	*95% CI*	*p-value*
(Intercept)	0.00	0.00 – 0.00	**<0.001**
Euclidean distance from roads	0.00	0.00 – 0.00	**<0.001**
Elevation (meters above sea level)	0.00	0.00 – 0.08	**0.001**
Distance from the sea (m)	9.16	2.43 – 33.43	**0.001**
Maximum temperature of warmest month, 1970 - 2000 (°C)	0.00	0.00 – 0.05	**<0.001**
Precipitation of the wettest month, 1970 - 2000 (mm)	0.16	0.09 – 0.29	**<0.001**
Distance from bush forest (secondary) (m)	1.51	0.93 – 2.44	0.094
Distance from old forest (primary) (m)	1.35	1.07 – 1.69	**0.010**

Supplemental Table 4. Multivariate environmental risk factor analysis of poultry distribution. Adjusted odds ratios for fixed effects which impact the odds poultry ownership. The model with lowest AIC is shown (68102). The log likelihood was --34034.95. MASS::stepAIC was used to determine the model with the lowest AIC using a stepwise parsimonious approach (both forward and backward selection)[6]. Data was mean centred and scaled prior to analysis. Odds ratios were calculated and plotted using the sjPlot[7] package in RStudio[8,9]. 95% confidence intervals (CI) indicate uncertainty around the adjusted odds ratios, while p-values indicate the statistical significance of the odds ratio (i.e. whether inclusion of the predictor variable significantly impacts the odds of poultry ownership). Bold p-values indicates that inclusion of a variable is statistically significant with p <0.05. P-values for the risk factors for the odds of poultry ownership are as follows $<2x10^{-16}$ (model intercept), $<2x10^{-16}$ (Euclidean distance from roads), 0.008 (population density per km^2), $2.27x10^{-6}$ (elevation in meters above sea level), 0.011 (aspect in degrees), $<2x10^{-16}$ (distance from the sea in meters), $<2x10^{-16}$ (average temperature in °C), 0.002 (mean diurnal range in °C), 0.001 (maximum temperature of warmest month in °C), $<2x10^{-16}$ (minimum temperature of coldest month in °C), $<2x10^{-16}$ (precipitation of the wettest month in mm), $<2x10^{-16}$ (distance from bush forest in metres), 0.0003 (distance from mangroves in meters), 0.045 (distance from oil palm plantations in meters), and 0.028 (distance from irrigated farmland in meters).

Environmental risk factors	Odds of poultry ownership		
Predictors	*Adjusted odds ratios*	*95% CI*	*p-value*
(Intercept)	0.59	0.58 – 0.59	**<0.001**
Euclidean distance from roads	0.98	0.98 – 0.98	**<0.001**
Population density (per km^2)	0.99	0.99 – 1.00	**0.008**
Elevation (meters above sea level)	1.10	1.06 – 1.14	**<0.001**
Aspect (degrees)	0.99	0.99 – 1.00	**0.011**
Distance from the sea (m)	0.78	0.75 – 0.81	**<0.001**
Average temperature, 1970 - 2000 (°C)	3.49	2.54 – 4.79	**<0.001**
Mean diurnal range, 1970 - 2000 (°C)	1.10	1.04 – 1.17	**0.002**
Maximum temperature of warmest month, 1970 - 2000 (°C)	0.82	0.73 – 0.92	**0.001**
Minimum temperature of coldest month, 1970 - 2000 (°C)	0.31	0.25 – 0.39	**<0.001**
Precipitation of the wettest month, 1970 - 2000 (mm)	1.03	1.03 – 1.04	**<0.001**
Distance from bush forest (secondary) (m)	0.96	0.95 – 0.97	**<0.001**
Distance from mangroves (m)	0.95	0.93 – 0.97	**<0.001**
Distance from oil palm plantation (m)	1.01	1.00 – 1.01	**0.045**
Distance from irrigated farmland (m)	0.99	0.99 – 1.00	**0.028**

Distance from old forest (primary) (m)	1.03	1.02 – 1.03	**<0.001**

Supplemental Table 5. Models of wild shorebird distribution and H5 binding with null, separate, and joint spatial effects.

**Model**		**DIC**	**Marginal likelihood**	**Spatial range (km)**
M1	No spatial or environmental effect (H5)	8762.31	-4381.26	
M2	Spatial effect only (H5)	388.69	-201.07	47.2769
M3	Spatial and environmental effect (H5)	378.30	-200.56	28.7355
M4	No spatial or environmental effect (wild birds)	1526.31	-763.15	
M5	Spatial effect only (wild birds)	5104.99	-184.94	94.7576
M6	Spatial and environmental effect (wild birds)	1098.52	-173.29	87.1406
M7	Combined model of wild birds and H5, separate spatial effect	1454.16	-368.85	1.0517
M8	Combined model of wild birds and H5, shared spatial effect	1410.58	-374.13	0.879029<br><br>Scaling parameter: 1.058

Supplemental Table 6. Covariates in model of H5 binding with shorebird distribution and shared spatial effects.

**Probability of H5 binding**	**Mean**	**SD**	**95% BCI**
Normalized differential vegetation index	0.627	0.236	0.164, 1.089
Elevation (meters above sea level)	-2.317	1.989	-6.214, 1.581
Distance from the sea (m)	1.069	2.137	-3.119, 5.257
Mean diurnal range, 1970 - 2000 (°C)	-0.353	2.127	-4.522, 3.816
Minimum temperature of coldest month, 1970 - 2000 (°C)	-3.570	2.261	-8.001, 0.862
Precipitation of the wettest month. 1970 - 2000 (mm)	-0.707	0.851	-2.375, 0.961
Precipitation seasonality, 1970 - 2000 (coefficient of variation)	1.356	1.852	-2.274, 4.987
Distance from irrigated farmland (m)	-1.293	0.940	-3.134, 0.549
**Probability of shorebird sightings**			
Euclidean distance from roads	-6.364	1.939	-10.164, -2.565
Elevation (meters above sea level)	-2.959	2.132	-7.138, 1.220
Distance from the sea (m)	0.612	2.716	-4.710, 5.935
Maximum temperature of warmest month, 1970 - 2000 (°C)	0.754	2.055	-3.275, 4.782

Precipitation of the wettest month, 1970 - 2000 (mm)	0.882	1.845	-2.734, 4.498
Distance from bush forest (secondary) (m)	1.458	0.550	0.381, 2.536
Distance from old forest (primary) (m)	0.985	1.194	-1.356, 3.326

Supplemental Table 7. Inclusion of poultry and swine ownership in multivariate environmental risk factor analysis of H5N1 binding distribution**.** Adjusted odds ratios for fixed effects which impact the odds of H5 binding being greater than 2 AU. These covariates were added to the model in **Supplemental Table 2** and compared to the previous model by likelihood ratio tests. Data was mean centred and scaled prior to analysis. Odds ratios were calculated and plotted using the sjPlot[7] package in RStudio[8,9].

	Adjusted odds ratios	*95% CI*	*Covariate p-value*	*Model comparison p-value (by likelihood ratio tests) to Supplemental Table 2*
Poultry ownership	0.96	0.83-1.09	0.580	0.319
Swine ownership	1.09	0.71 – 1.39	0.592	0.247

Supplemental Figure 6. Distribution of poultry ownership per village. Figure generated in QGIS 3.30.2[2]. Natural Earth raster map data was used to generate the canvas map.

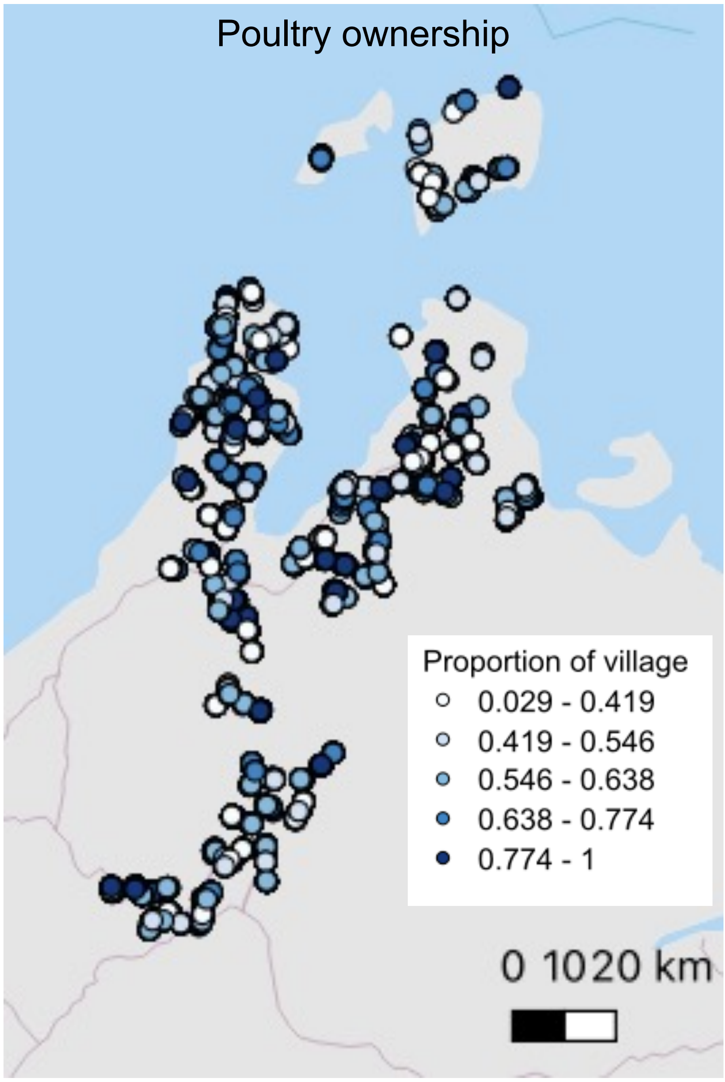

Supplemental Figure 7. Distributions of waterfowl and shorebirds sightings in Sabah. Abundance of species observed in the Sabah region of Malaysian Borneo from July 1979 to August 2023 and reported to the eBird database. Waterfowl species included wandering whistling duck (*Dendrocygna arcuata),* lesser whistling duck (*Dendrocygna javanica*), tufted duck (*Aythya fuligula*), garganey (*Spatula querquedula*), northern shoveler (*Spatula clypeata*), Eurasian wigeon (*Mareca penelope*), Chinese spot-billed duck (*Anas zonorhyncha*), mallard duck (*Anas platyrhynchos*), grey teal (*Anas gibberifrons*), northern pintail (*Anas acuta*), common teal (*Anas crecca*), Asian pygmy goose (*Nettapus coromandelianus*). Shorebird species are listed in **Table 1**. Figure generated in QGIS 3.30.2[2]. Natural Earth raster map data was used to generate the canvas map.

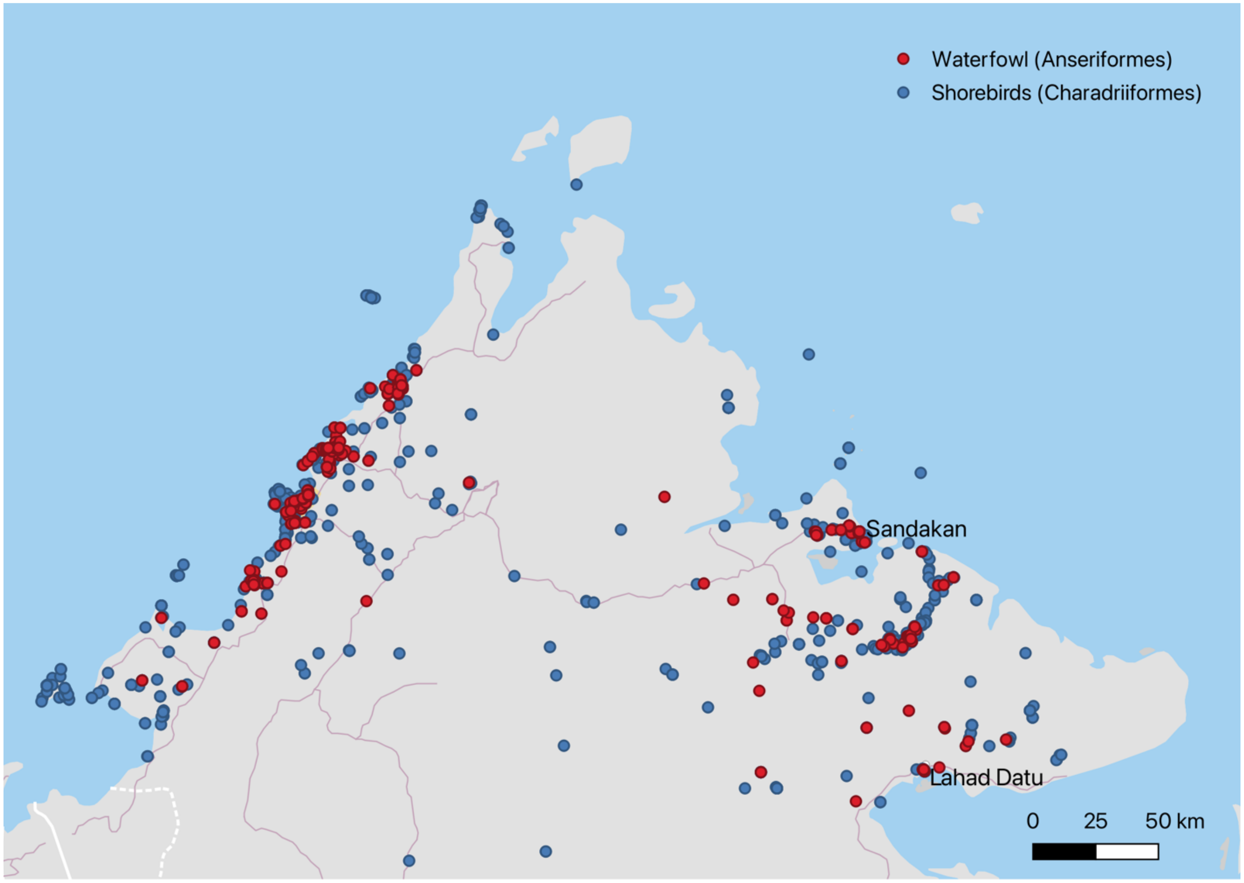

Supplemental Figure 8. Pearson correlation for environmental variables included in this study. Highly correlated variables with Pearson's correlation coefficients > 0.8 were not included in final environmental models, as in Fornace et al[1].

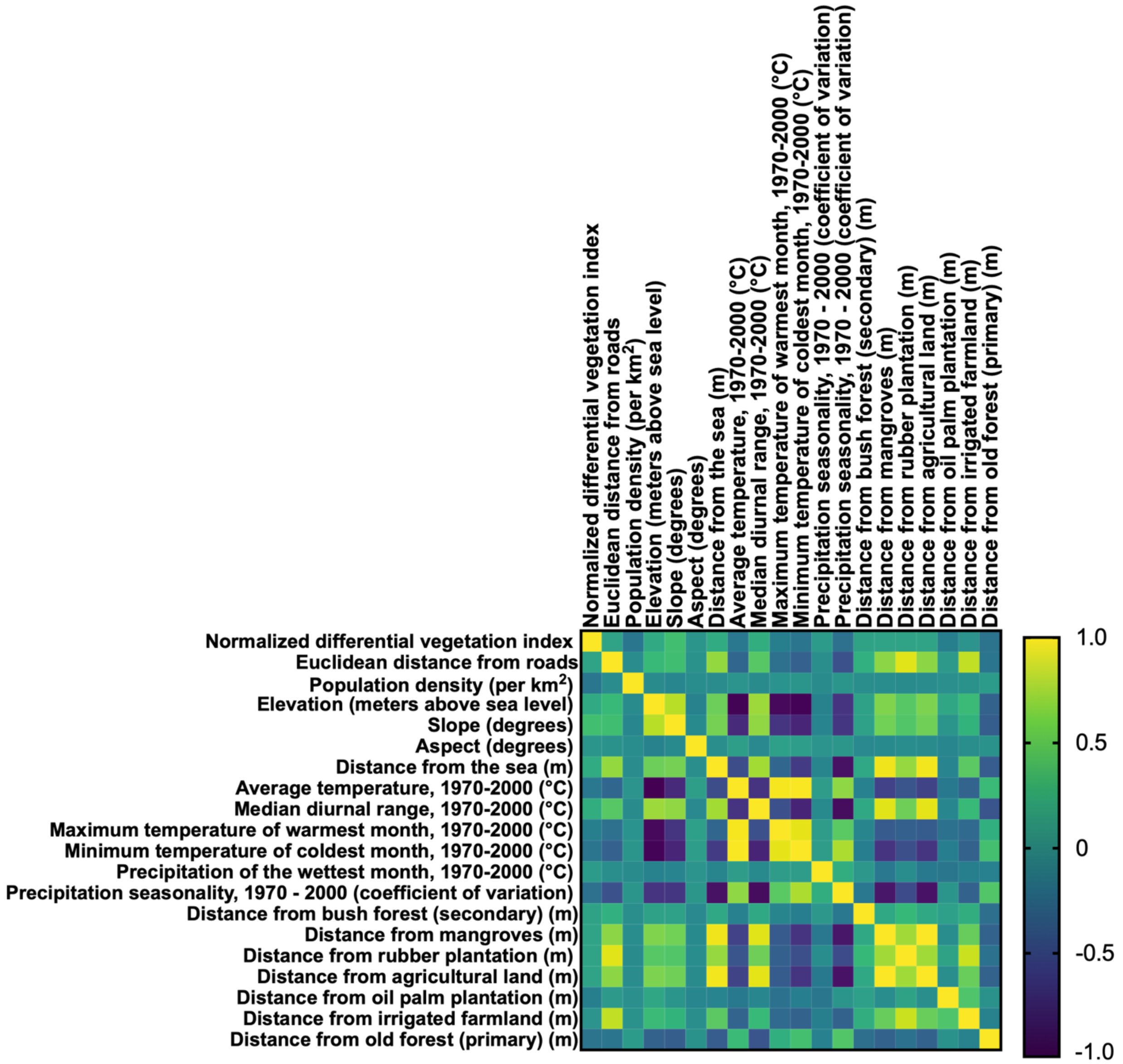

8. Viral proteins, antibodies, & antiserum

Supplemental Table 7. Viral proteins for ELISAs and bead-based assays.

**Protein**	**Strain**	**Catalog number; lot number(s)**	**Contributor**	**Purification method & tag**
H5 HA from H5N1	A/Duck/Laos/3295/2006	NR-13509; 61942042	St. Jude Children's Research Hospital (CEIRS)	“Purified by conventional chromatographic methods”
H5 HA from H5N2	A/snow goose/Missouri/CC15-84A/2015	NR-50651; 70003601	St. Jude Children's Research Hospital (CEIRS)	“Purified by conventional chromatographic methods”
H3 HA from H3N2	A/New York/55/2004	NR-19241; 59311910	BEI Resources	“Purified under conditions that preserve its biological activity and tertiary structure”
H3 HA from H3N2	A/Wisconsin/67/2005	NR-49237; 70052281	BEI Resources	Nickel affinity chromatography & C-terminal histidine tag
H1 HA from H1N1 pdm09	A/California/07/2009	NR-51668; 70026141	St. Jude Children's Research Hospital	Ion exchange and affinity chromatography
H1 HA from H1N1 pdm09	A/New York/18/2009	NR-51159; 70019462	BEI Resources	Affinity chromatography & C-terminal histidine tag
H1 HA from H1N1 pdm09	A/California/04/2009	NR-15749; 70041927	BEI Resources	Nickel affinity chromatography & C-terminal histidine tag

Supplemental Table 8. Antibodies and antiserum for ELISAs. PBMCs stands for peripheral blood mononuclear cells.

Reagent	Details	Catalog number; lot number	Provider	Contributor	Production information
Polyclonal goat antiserum H5 HA	A/Duck/Laos/3295/2006 (H5N1)	NR-18931; 59137401	BEI Resources	St. Jude Children's Research Hospital (CEIRS)	Immunization of a goat with baculovirus-expressed HA
Polyclonal goat antiserum H5 HA	A/snow goose/MO/CC15-84A/2015 (H5N1)	NR-50875; 70008201	BEI Resources	St. Jude Children's Research Hospital (CEIRS)	Immunization of a goat with bromelain-released HA
Polyclonal goat antiserum H5 HA	A/Bar Headed Goose/Qinghai/1A/05 (H5N1)	NR-14818; 58720709	BEI Resources	St. Jude Children's Research Hospital (CEIRS)	Immunization of a goat with baculovirus-expressed HA
Low titre human antisera H5N1	A/Indonesia/05/2005 (H5N1)	NR-33667; 60864514	BEI Resources	NIAID/NIH	Pooled polyclonal antisera from humans immunized intramuscularly with two doses of an inactivated monovalent subvirion investigational influenza vaccine: A/Indonesia/05/2005 PR8-IBCDC-RG2.
Medium titre human antisera H5N1	A/Indonesia/05/2005 (H5N1)	NR-33668; 60898956	BEI Resources	NIAID/NIH	
Polyclonal goat antiserum H1 HA	A/California/04/2009 (H1N1) (antiserum, goat)	NR-15696; 58895528	BEI Resources	St. Jude Children's Research Hospital (CEIRS)	Immunization of a goat with baculovirus-expressed HA

High titre human convalescent sera H1N1	2009 H1N1 (strain not provided)	NR-18964; 59164848	BEI Resources	NIAID/NIH	Convalescent serum collected post infection. High and low indicate relative antibody titres according to the provider.
Low titre human convalescent sera H1N1	2009 H1N1 (strain not provided)	NR-18965; 59163837	BEI Resources	NIAID/NIH	
Polyclonal goat antiserum H3 HA	A/Hong Kong/2286/2017 (H3N2)	NR-51700; 70027287	BEI Resources	NIAID/NIH	Immunization of a goat with bromelain-released HA
Secondary antibody, anti-goat	Donkey anti-Goat IgG (H+L) Cross-Adsorbed Secondary Antibody, HRP	A16005; 70-18-04-2419 & 77-56-060528	Thermo Fisher Scientific	Invitrogen	
Secondary antibody, anti-human IgG	Anti-Human IgG (Fc specific) −Peroxidase antibody produced in goat	A0170-1ML; 0000129825	Merck Life Science UK Limited	Sigma-Aldrich	

9. Pseudovirus titration results

Supplemental Figure 9. Pseudotyped influenza titration results. Titration data collected for the pseudoviruses used in this study A/Indonesia/05/2005 (Clade 2.1)(n=10 preparations), A/Bar headed goose/Qinghai/1A/2005 (Clade 2.2)(n=10 preparations), A/chicken/Malaysia(Sabah)/6123/2018 (Clade 2.3.2.1c)(n=8 preparations). Preparations of pseudovirus were titrated in duplicate and pooled for use in neutralisation assays. The minimum relative light units (RLU) accepted for use in this assay was 1*10^5. Data was analysed and graphed in GraphPad Prism 10.0.3.

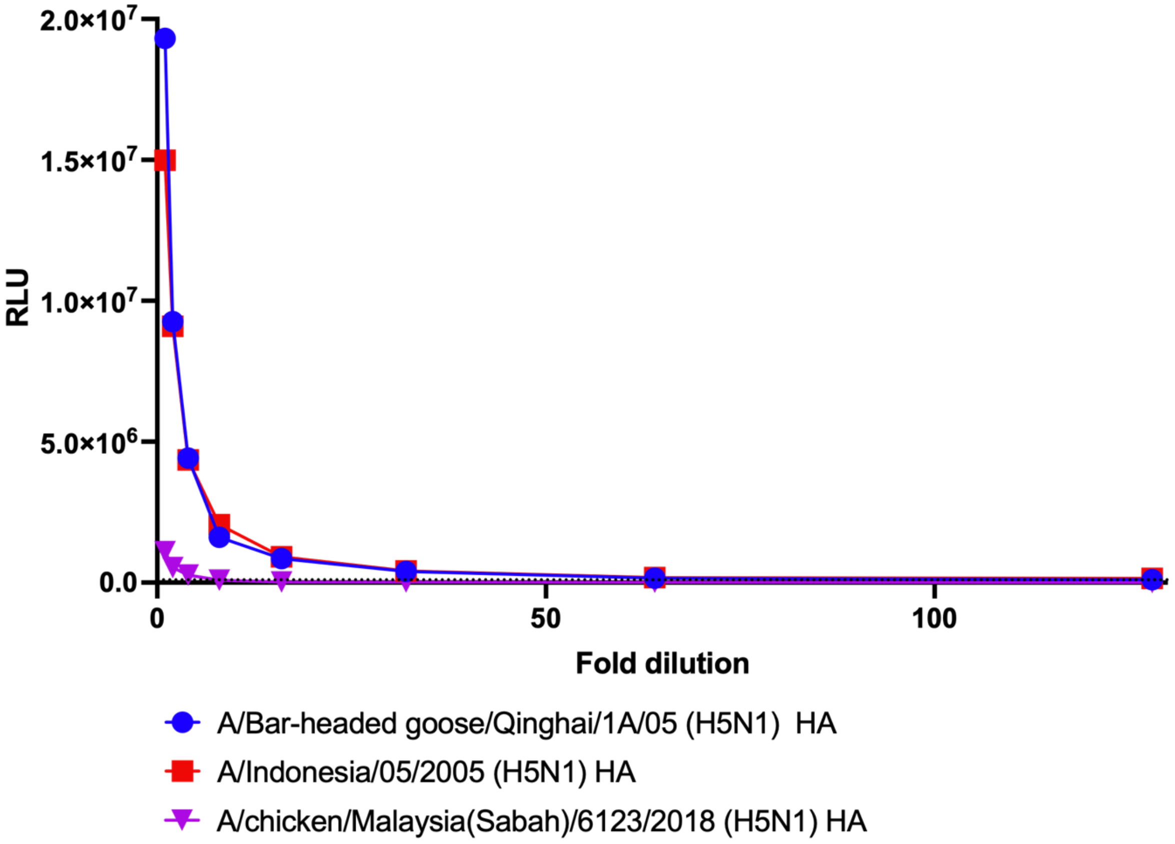

10. Cross-reactivity depletion assay optimisation

Supplemental Table 9. Buffers required for cross-reactivity depletion assay.

**Buffer name**	**Components (*supplier, product code*)**
Block buffer	1. 500mL 1X PBS<br>2. 0.1% Bovine serum albumin (*Sigma-Aldrich, B6917-100MG)*<br>3. 0.02% Tween® 20<br>4. 0.05 % Sodium Azide *(Sigma-Aldrich, 71289-50G)*
Wash buffer	1. 500mL 1X PBS<br>2. 0.1% Bovine serum albumin<br>3. 0.02 % Tween® 20
Storage buffer	1. 500mL 1X PBS<br>2. 0.05% sodium azide
Assay buffer	1. 500mL 1X PBS<br>2. 0.1% Bovine serum albumin

Supplemental Figure 10. Optimisation of bead-based cross reactivity depletion assay. a. Effect of successive bead passages on pseudotyped neutralisation. The original NT50 of medium titre human convalescent H1N1 sera (BEI Resources) was 2662 against A/Indonesia/05/2005(H5N1) HA. After five passages of fresh beads diluted to 300 beads/uL (cocktail mixture) NT50 dropped to 349.8, which was statistically significant $p<0.0001$ by a sum-of-squares F Test in GraphPad. After 10 passages, the reduction in NT50 was not statistically significant (NT50=251.2, $p=0.1205$). Against A/Bar-headed goose/Qinghai/1A/05 (H5N1) HA, the NT50 for this sample was 1421, after five passages this was 540.7 ($p=0.0003$), and after 10 passages this was 535.5 ($p=0.9749$). This suggests that five passages was the optimal choice to preserve beads, virus, and plasma. **b.** Effect of non-coated beads on pseudotyped neutralisation. The NT50 of low titre human antisera H5N1 (BEI Resources) was 3862 against A/Bar-headed goose/Qinghai/1A/05 (H5N1) HA, after five passages of beads which were not coated with any HA, the NT50 did not change statistically significantly (NT50=4257, $p=0.8490$). However, when treated with seasonal HA beads, the NT50 of this plasma sample experienced a statistically significant reduction (NT50=1441, $p=0.0016$). **c.** Effect of successive bead passages on binding to seasonal and avian HAs as tested by ELISAs. Before and after five passages of fresh beads diluted to 300 beads/uL (cocktail mixture), binding of medium titre human antisera H5N1 and medium titre human convalescent H1N1 sera (BEI Resources) to H1N1, H3N2, and H5N1 HA was tested in duplicate. These samples are referred to as negative control 2 and postive pool 1 in Figure 3a. Pre and post treatment binding values are displayed on the graphs.

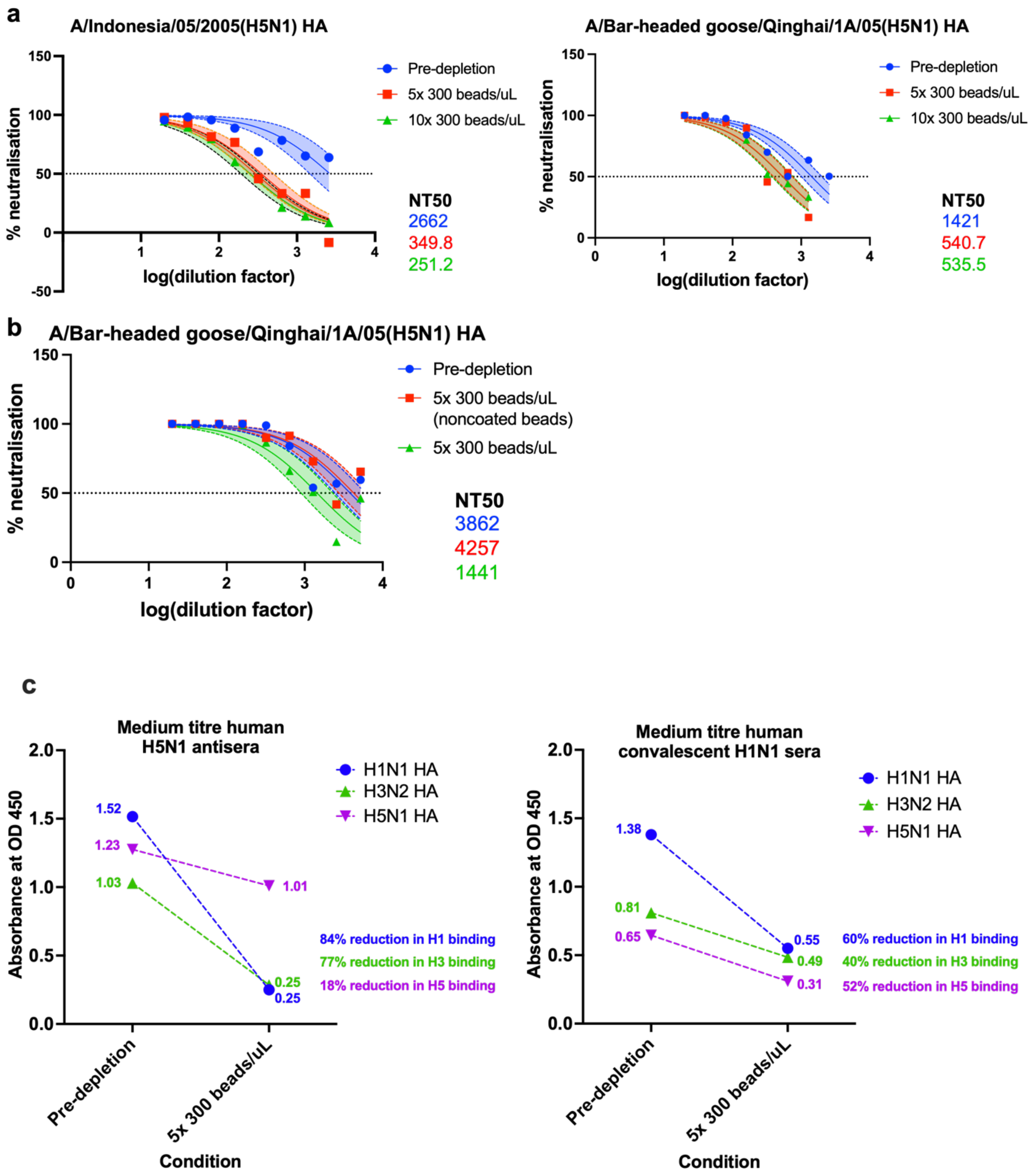
a
A/Indonesia/05/2005(H5N1) HA
% neutralisation
log(dilution factor)
Pre-depletion
5x 300 beads/uL
10x 300 beads/uL
NT50
2662
349.8
251.2
A/Bar-headed goose/Qinghai/1A/05(H5N1) HA
% neutralisation
log(dilution factor)
Pre-depletion
5x 300 beads/uL
10x 300 beads/uL
NT50
1421
540.7
535.5
b
A/Bar-headed goose/Qinghai/1A/05(H5N1) HA
% neutralisation
log(dilution factor)
Pre-depletion
5x 300 beads/uL (noncoated beads)
5x 300 beads/uL
NT50
3862
4257
1441
c
Medium titre human H5N1 antisera
Absorbance at OD 450
Condition
Pre-depletion
5x 300 beads/uL
H1N1 HA
H3N2 HA
H5N1 HA
1.52
1.23
1.03
1.01
0.25
0.25
84% reduction in H1 binding
77% reduction in H3 binding
18% reduction in H5 binding
Medium titre human convalescent H1N1 sera
Absorbance at OD 450
Condition
Pre-depletion
5x 300 beads/uL
H1N1 HA
H3N2 HA
H5N1 HA
1.38
0.81
0.65
0.55
0.49
0.31
60% reduction in H1 binding
40% reduction in H3 binding
52% reduction in H5 binding

Supplementary references

1 Fornace, K. M. *et al.* Environmental risk factors and exposure to the zoonotic malaria parasite Plasmodium knowlesi across northern Sabah, Malaysia: a population-based cross-sectional survey. *The Lancet Planetary Health* **3**, e179-e186, doi:10.1016/s2542-5196(19)30045-2 (2019).

2 QGIS Geographic Information System v. 3.30.2 (Open Source Geospatial Foundation, 2009).

3 Exchange;, H. D. in *COD - Subnational Administrative Boundaries* (ed United Nations Office for the Coordination of Humanitarian Affairs) (2023).

4 Thompson, C. P. *et al.* Detection of neutralising antibodies to SARS-CoV-2 to determine population exposure in Scottish blood donors between March and May 2020. *Eurosurveillance* **25**, doi:10.2807/1560-7917.es.2020.25.42.2000685 (2020).

5 Klim, H. *et al.* Quantifying human-animal contact rates in Malaysian Borneo: Influence of agricultural landscapes on contact with potential zoonotic disease reservoirs. *Frontiers in Epidemiology* **2**, doi:10.3389/fepid.2022.1057047 (2023).

6 Venables, W. & Ripley, B. *Modern Applied Statistics with S*. 4th edn, (Springer, 2002).

7 **sjPlot: Data Visualization for Statistics in Social Science** v. 2.8.15 (2023).

8 R Core Team. *R: A language and environment for statistical computing.* , <https://www.R-project.org/.> (2020).

9 RStudio: Integrated Development for R. (RStudio, PBC, Boston, MA, 2020).
