# Supplementary material for: Serological analysis in humans in Malaysian Borneo suggests prior exposure to H5 avian influenza near migratory shorebird habitats
Source: Nat Commun. 2024 Oct 17;15:8863. doi: 10.1038/s41467-024-53058-y (PMC11487116; doi:10.1038/s41467-024-53058-y)
Supplement: Supplementary file 3 — Reporting Summary [file 41467_2024_53058_MOESM3_ESM.pdf]

nature portfolio

Corresponding author(s): Hannah Klim, Kimberly Fornace

Last updated by author(s): Sep 16, 2024

Reporting Summary

Nature Portfolio wishes to improve the reproducibility of the work that we publish. This form provides structure for consistency and transparency in reporting. For further information on Nature Portfolio policies, see our Editorial Policies and the Editorial Policy Checklist.

Statistics

For all statistical analyses, confirm that the following items are present in the figure legend, table legend, main text, or Methods section.

n/a	Confirmed	
☐	☒	The exact sample size (*n*) for each experimental group/condition, given as a discrete number and unit of measurement
☐	☒	A statement on whether measurements were taken from distinct samples or whether the same sample was measured repeatedly
☐	☒	The statistical test(s) used AND whether they are one- or two-sided<br>*Only common tests should be described solely by name; describe more complex techniques in the Methods section.*
☐	☒	A description of all covariates tested
☐	☒	A description of any assumptions or corrections, such as tests of normality and adjustment for multiple comparisons
☐	☒	A full description of the statistical parameters including central tendency (e.g. means) or other basic estimates (e.g. regression coefficient) AND variation (e.g. standard deviation) or associated estimates of uncertainty (e.g. confidence intervals)
☐	☒	For null hypothesis testing, the test statistic (e.g. *F*, *t*, *r*) with confidence intervals, effect sizes, degrees of freedom and *P* value noted<br>*Give P values as exact values whenever suitable.*
☐	☒	For Bayesian analysis, information on the choice of priors and Markov chain Monte Carlo settings
☐	☒	For hierarchical and complex designs, identification of the appropriate level for tests and full reporting of outcomes
☐	☒	Estimates of effect sizes (e.g. Cohen's *d*, Pearson's *r*), indicating how they were calculated

Our web collection on statistics for biologists contains articles on many of the points above.

Software and code

Policy information about availability of computer code

Data collection	Sample collection is described in previously published papers. All data generating activities (e.g. lab analysis) and use of open-source data (e.g. wild bird sightings) are described in the manuscript.
Data analysis	The data was analysed in RStudio 4.2.0, GraphPad Prism 10.0.3, and QGIS 3.30.2 as noted in the text. All code produced in R for this study is available at: https://github.com/hklim06/Serological-analysis-in-humans-in-Malaysian-Borneo-suggests-prior-exposure-to-H5-avian-influenza.

For manuscripts utilizing custom algorithms or software that are central to the research but not yet described in published literature, software must be made available to editors and reviewers. We strongly encourage code deposition in a community repository (e.g. GitHub). See the Nature Portfolio guidelines for submitting code & software for further information.

Data

Policy information about availability of data

All manuscripts must include a data availability statement. This statement should provide the following information, where applicable:

- Accession codes, unique identifiers, or web links for publicly available datasets
- A description of any restrictions on data availability
- For clinical datasets or third party data, please ensure that the statement adheres to our policy

The environmental data generated in this study have been deposited on HK’s GitHub under accession code [https://doi.org/10.5281/zenodo.13767296]. All environmental data was curated as in Klim et al. and Fornace et al.. 20 environmental variables were considered for inclusion in the final models of shorebird contact and H5 binding. Elevation, aspect, and slope data for the region were obtained from the ASTER Digital Global Elevation Model55. The WorldClim database

was used to collect data from 1970-2000 on average temperature, minimum temperature of coldest month, maximum temperature of warmest month, mean diurnal range (all in °C), precipitation seasonality (coefficient of variation), precipitation of wettest month (mm), and population density (per km2). Household distance in meters from mangroves, agricultural land, irrigated farmland, the sea, old (primary) forest, bush (secondary) forest, oil palm plantations, rubber plantations, and Euclidean distance from roads were calculated by Fornace et al.

The survey and serological data are available under restricted access. As the data includes identifiable information and household coordinates, data can be obtained with approval from relevant ethics committees in Malaysia and the UK. Please contact corresponding authors for further details.

Research involving human participants, their data, or biological material

Policy information about studies with human participants or human data. See also policy information about sex, gender (identity/presentation), and sexual orientation and race, ethnicity and racism.

Reporting on sex and gender	This study collected data on self-reported gender; however, our analysis does not focus on sex or gender. Both male and female participants were included in the study. We do not make conclusions based on sex or gender.
Reporting on race, ethnicity, or other socially relevant groupings	We did not perform analysis based on race, ethnicity, or socio-economic status in this study.
Population characteristics	The study population is a rural population from Sabah Malaysia surveyed in 2015. The two control sample populations include blood donors from Kota Kinabalu, Malaysia and Scottish blood donors.
Recruitment	This survey was a two-stage randomly stratified cross-sectional survey. All participants were randomly selected for inclusion and written informed consent/ assent was obtained by local field staff. Full details of study recruitment are available in Fornace et. al, 2019, Lancet Planetary Health.
Ethics oversight	Ethical approvals were obtained for this study from the Malaysian Ministry of Health, the Research Ethics Committee of the London School of Hygiene & Tropical Medicine, and the Oxford Tropical Research Ethics Committee. Use of the background cohorts (Scottish Blood donors and Kota Kinabalu) were obtained from the Scottish National Blood Transfusion Service and the Ethics Committee of the Faculty of Medicine and Health Sciences, Universiti Malaysia Sabah, respectively.

Note that full information on the approval of the study protocol must also be provided in the manuscript.

Field-specific reporting

Please select the one below that is the best fit for your research. If you are not sure, read the appropriate sections before making your selection.

☒ Life sciences ☐ Behavioural & social sciences ☐ Ecological, evolutionary & environmental sciences

For a reference copy of the document with all sections, see nature.com/documents/nr-reporting-summary-flat.pdf

Life sciences study design

All studies must disclose on these points even when the disclosure is negative.

Sample size	From 10,100 total individuals included in the cross-sectional survey (rural Malaysian cohort), 2000 were randomly selected for this study. We additionally screened 678 samples from blood donors in Kota Kinabalu (urban Malaysian cohort) and 63 Scottish blood donor samples, which were used as control/ background samples. For the Kota Kinabalu and Scottish blood donor samples, all available samples were tested (n=678 and n=63, respectively). Sample size calculations for the collection of the rural Malaysian samples were performed by Fornace et al (Lancet Planetary Health, 2019). 2000 samples were selected with 500 from each geographic region (to ensure an even spatial distribution of samples), as it was not possible to test all 10,100 samples by ELISA due to resource constraints. As there is no available data on expected seroprevalence for H5 in this region, we could not estimate the underlying rate of H5 exposure in the population for a sample size calculation. We instead opted to test as many samples as was feasible on all ELISA antigens.
Data exclusions	No data were excluded from the analyses.
Replication	All assays were performed with at least two replicates, as noted throughout the study. All attempts at replication were successful.
Randomization	500 samples from each geographic district (n=4) in the study were randomly selected for serological testing.
Blinding	Investigators were aware of which sample collection the samples came from (rural Malaysian, urban Malaysian, or Scottish blood donors). Blinding was not possible with the urban Malaysian samples, as these assays were run in Malaysia, while assays involving the rural Malaysian and Scottish blood donor samples were run in the UK. Investigators were therefore aware of which cohort samples belonged to. Blinding was additionally not necessary for this study, as all individuals were presumed to have likely previous exposure to influenza A virus. It was only through subsequent data analysis that investigators were able to determine that the rural Malaysian samples contained high H5 responses. Additionally, knowledge of the sample background was useful to investigators to determine baselines for PNA assays and true negatives or true positives for the cross-reactivity depletion assay optimization.

Reporting for specific materials, systems and methods

We require information from authors about some types of materials, experimental systems and methods used in many studies. Here, indicate whether each material, system or method listed is relevant to your study. If you are not sure if a list item applies to your research, read the appropriate section before selecting a response.

Materials & experimental systems

n/a	Involved in the study
☐	☒ Antibodies
☐	☒ Eukaryotic cell lines
☒	☐ Palaeontology and archaeology
☒	☐ Animals and other organisms
☒	☐ Clinical data
☒	☐ Dual use research of concern
☒	☐ Plants

Methods

n/a	Involved in the study
☒	☐ ChIP-seq
☐	☒ Flow cytometry
☒	☐ MRI-based neuroimaging

Antibodies

Antibodies used	All antibodies and antiserum including catalog numbers, lot numbers, provider information, and production information is provided in detail in Supplemental Table 8 of the supplemental information included with this submission. For ELISAs both anti-goat (Catalog number: A16005, Lot numbers: 70-18-04-2419 & 77-56-060528, ThermoFisher Scientific) and anti-human antibodies (Catalog number: A0170, Lot number: 0000129825, Merck Life Science UK Limited) were used at 1:2000 (as recommended by the suppliers and shown by our colleagues in Bore et al, Nature Communications, 2024).
Validation	All antibodies were validated by the manufacturers as described.

Eukaryotic cell lines

Policy information about cell lines and Sex and Gender in Research

Cell line source(s)	HEK 293T cell line (Sigma, 12022001)
Authentication	Original Molecular assessment from supplier but not repeated in Lab
Mycoplasma contamination	Regular mycoplasma screening performed, all cell lines tested negative.
Commonly misidentified lines (See ICLAC register)	No commonly misidentified cell lines were used in this study.

Plants

Seed stocks	*Report on the source of all seed stocks or other plant material used. If applicable, state the seed stock centre and catalogue number. If plant specimens were collected from the field, describe the collection location, date and sampling procedures.*
Novel plant genotypes	*Describe the methods by which all novel plant genotypes were produced. This includes those generated by transgenic approaches, gene editing, chemical/radiation-based mutagenesis and hybridization. For transgenic lines, describe the transformation method, the number of independent lines analyzed and the generation upon which experiments were performed. For gene-edited lines, describe the editor used, the endogenous sequence targeted for editing, the targeting guide RNA sequence (if applicable) and how the editor was applied.*
Authentication	*Describe any authentication procedures for each seed stock used or novel genotype generated. Describe any experiments used to assess the effect of a mutation and, where applicable, how potential secondary effects (e.g. second site T-DNA insertions, mosiacism, off-target gene editing) were examined.*

Flow Cytometry

Plots

Confirm that:

- [x] The axis labels state the marker and fluorochrome used (e.g. CD4-FITC).
- [x] The axis scales are clearly visible. Include numbers along axes only for bottom left plot of group (a 'group' is an analysis of identical markers).
- [ ] All plots are contour plots with outliers or pseudocolor plots.
- [x] A numerical value for number of cells or percentage (with statistics) is provided.

Methodology

Sample preparation	Carboxyl magnetic particles (beads) were conjugated with IAV HAs, as described in the methods section of the manucript. All previously prepared beads were diluted to 200beads/uL and incubated with control serum. Beads were washed and incubated with phycoerythrin (PE)-conjugated anti-IgG (Southern Biotech #9040-09) or isotype control (BioLegend #400112) secondary antibody (described in detail in Methods section).
Instrument	LSRFortessa™ X-20 was used for data collection.
Software	Data was analysed in FlowJo™ 10.8.2.
Cell population abundance	N/A.
Gating strategy	Side scatter versus forward scatter plots were used to draw gate 1 to exclude debris and doublets. The count versus PE histogram was used to observe PE fluorescence in the different conditions (Supplemental Figure 3). Gating strategy figure for beads can be added to SI if needed.

- [x] Tick this box to confirm that a figure exemplifying the gating strategy is provided in the Supplementary Information.
